# Impact of Coronavirus Infectious Disease (COVID-19) pandemic on willingness of immunization—A community-based questionnaire study

**DOI:** 10.1371/journal.pone.0262660

**Published:** 2022-01-14

**Authors:** Pei-Yun Chen, Pei-Ni Chuang, Chien-Hsieh Chiang, Hao-Hsiang Chang, Chia-Wen Lu, Kuo-Chin Huang

**Affiliations:** 1 Department of Family Medicine, National Taiwan University Hospital Bei-Hu Branch, Taipei, Taiwan; 2 Department of Family Medicine, National Taiwan University Hospital, Taipei, Taiwan; 3 Training Center for Travel Medicine, National Taiwan University Hospital, Taipei, Taiwan; 4 Department of Family Medicine, National Taiwan University BioMedical Park Hospital, Hsin-Chu, Taiwan; Uniwersytet Zielonogorski, POLAND

## Abstract

**Background:**

Coronavirus infectious disease 2019 (COVID-19) has had a great impact on global health, but with relatively few confirmed cases in Taiwan. People in Taiwan showed excellent cooperation with the government for disease prevention and faced social and behavioral changes during this period. This study aimed to investigate people’s knowledge of COVID-19, attitudes and practices regarding vaccinations for influenza, pneumococcus and COVID-19.

**Methods:**

We conducted a community-based, cross-sectional questionnaire survey from September 2020 to October 2020 among adults in northern Taiwan. The four-part questionnaire included questions on sociodemographic characteristics, knowledge, attitude, and practice toward COVID-19.

**Results:**

Among a total of 410 respondents, 58.5% were categorized as having “good knowledge” responding to COVID-19. Among the total respondents, 86.6% were willing to receive influenza or pneumococcal vaccines, and 76% of them acted to receive COVID-19 immunization once the vaccine became available. Compared with the respondents with poor knowledge of COVID-19, those with good knowledge had a more positive attitude toward receiving influenza or pneumococcal immunization (OR 3.26, 95% CI = 1.74–6.12).

**Conclusions:**

Participants with good knowledge of COVID-19 had greater intent to receive immunization for influenza or pneumococcal vaccine. The promotion of correct knowledge of both COVID-19 and immunization preparations is necessary.

## Introduction

### Background

The pandemic coronavirus infectious disease (COVID-19) caused by severe acute respiratory syndrome coronavirus 2 (SARS-CoV-2) emerged from Wuhan, China, in December 2019 and has spread widely across the world. Taiwan is geographically near mainland China and was presumed to have high numbers of COVID-19 cases [1]. However, the disease was not prevalent in Taiwan due to the contributions of brisk responses by the government to disease control and cooperation from the general population of Taiwan [2]. Information about the disease entity, the route of transmission and the preventive guidelines were updated soon after the COVID-19 outbreak [3–5]. The Taiwan Centers for Disease Control (Taiwan CDC) updated the population regarding the confirmed cases and quarantine policies daily and educated the public to ensure they had access to correct information about self-hygiene and disease prevention from the very beginning [6]. Citizens with mobile devices can easily link to resources and obtain recent and correct information. In addition, COVID-19 confirmed case number updating, policy renewals, such as the required duration for quarantine, and disease prevention guidelines, such as social distancing and hand washing, were also announced on these social media platforms. Thus, citizens could instantly capture first-hand and transparency data from the Taiwan CDC [7]. In comparison with the worldwide COVID-19 pandemic, which reached 77.5 million people infected with a mortality of approximately 1.7 million, there were 770 cumulative confirmed cases, mostly imported, and 7 deaths reported in Taiwan as of December 22^nd^, 2020 [8, 9].

Similar to COVID-19, influenza is a contagious and easily mutated RNA virus that needs to be protected against by vaccination annually but still causes seasonal epidemics. The World Health Organization (WHO) encourages countries to conduct routine influenza surveillance [10–12]. The Taiwanese government-funded annual program of seasonal influenza immunization initially supplied vaccines for high-risk groups, including those aged 65 and above or aged 50–64 with comorbidities. After expanding vaccine coverage, a government-funded influenza vaccine has been available for the healthy population aged 50–64 since 2016 [13, 14]. However, the immunization rates have been relatively low at 18.1% and 51.3% for those aged 50–64 and aged 65 and above, respectively [15]. Consequently, the incidence rate of severe influenza infection-related morbidities has been reported to range from 0.4 to 1.8 per 100,000 people annually in Taiwan in the past 5 years [16]. According to previously published studies, the COVID-19 pandemic caused medical preference changes in reproductive and colon cancer screening issues [17, 18]. Similarly, people in Taiwan showed great cooperation with the government for disease prevention, which resulted in personal, social and behavioral modifications during this period. Although vaccines designed to protect against COVID-19 have also been urgently investigated worldwide and are under human clinical trials [19–21], little is known about what variables might influence the willingness to receive vaccinations.

### Aims of this study

The aims of this study are (1) to investigate the level of Taiwanese knowledge responding to COVID-19, and (2) to determine if knowledge about COVID-19 is related to attitudes and practices toward vaccinations for influenza, pneumococcus and COVID-19.

## Methods

### Design

This was a community-based, cross-sectional questionnaire survey distributed to adults in the general population from September 2020 to October 2020 in northern Taiwan. Return and completion of the questionnaire represented the subject’s consent for participation. The questionnaire was completed anonymously, and the need for consent was waived by the institution’s ethics committee.

### Subjects

The inclusion criteria were age equal to or over 20 years old and willingness and ability to complete the questionnaire. The study was approved by the Research Ethics Committee at National Taiwan University Hospital (NTUH) in Taiwan (202008054W) before the study was conducted.

### Questionnaire

The four-part questionnaire included questions on sociodemographic characteristics, knowledge, attitudes, and practices regarding COVID-19. The questionnaire was pretested for face validity by a panel of five physicians with experience in the clinical practice of preventive medicine from NTUH. Furthermore, nine scholars and experts with diverse backgrounds in public health, medicine, and bioethical research fields were invited to test the content validity of the questionnaire before the study started. The nine experts were asked to verify the accuracy and degree of difficulty of the items in parts II to IV. After the process, we tailored the impact of COVID-19 to two separate items in part III of the questionnaire: the worldwide COVID-19 pandemic and the COVID-19 endemic in Taiwan. We also performed reliability analysis to test for internal consistency. Cronbach’s alpha was 0.92 for all items in the questionnaire, with values of 0.91 for the items of knowledge regarding COVID-19, 0.93 for the items for attitude toward COVID-19, and 0.79 for the items of subject’s practice responding to COVID-19.

The included sociodemographic characteristics were sex, age, education level, medical history, occupation, habit of exercise, preference for seeking medical support and information resources regarding COVID-19. The other three parts of the questionnaire included the following components:

Knowledge regarding COVID-19: This part examined the subject’s knowledge in three aspects as follows: (1) the disease pattern of COVID-19, including characteristics and transmission route of SARS-CoV-2, (2) self-hygiene and policies for disease prevention, including maintaining social distance and not taking public transportation when one is under quarantine, and (3) proper protective equipment use, including wearing a medical face mask and maintaining correct hand hygiene, and understanding the proper agents for disinfection, such as ethanol and hypochlorous acid. The participants’ scoring system used a five-point Likert scale, ranging from very poor (1 point) to poor (2 points), uncertain (3 points), good (4 points) and excellent (5 points). Higher scores indicated better understanding regarding the knowledge of COVID-19 and correct ways to maintain self-hygiene and protective equipment usage. Those with a score of 1 to 3 points were considered to have poor knowledge, and those with a score of 4 to 5 points were considered to have good knowledge to respond to COVID-19 in each item. Subjects with all items of good knowledge were regarded as those with good knowledge.

Attitudes toward COVID-19: This part examined the subject’s perceptions regarding (1) the severity of the pandemic or endemic conditions of COVID-19, (2) the importance of government policies for the general population, and (3) the willingness to take other kinds of vaccines, such as influenza or pneumococcal vaccines. The scoring system used two five-point Likert scales, one to evaluate the importance, ranging from “very unimportant” (1 point), to unimportant (2 points), no comment (3 points), important (4 points) and very important (5 points), and the other to examine the agreement, with descriptions ranging from strongly disagree (1 point), to disagree (2 points), no comments (3 points), agree (4 points) and strongly agree (5 points). Higher scores of agreement and importance indicated positive attitudes regarding the government policies for responding to COVID-19 and the willingness to have immunizations.

Practices responding to COVID-19: This part examined the effect of COVID-19 on subjects’ behavior modifications, including avoiding crowded areas, decreasing exercise, postponing or decreasing follow-up for acute or chronic diseases, changing preference sites for medical evaluation, washing hands more frequently than before, increasing electronic platform usage, and receiving COVID-19 immunization if vaccines are available in the future. The scoring system used a five-point Likert scale, ranging from “strongly disagree (1 point) to disagree (2 points), no comments (3 points), agree (4 points) and strongly agree (5 points). Higher scores of agreements indicated that the subject’s behaviors changed to greater extent in response to COVID-19.

### Statistical analysis

Data are presented as the mean±SD for continuous variables and number (percentage) for categorical variables. The Student’s t test and chi-square test were used to compare the sociodemographic variables and the age stratification for continuous variables and categorial variables respectively. Multivariate logistic regression analyses were used to compare the willingness for immunization between subjects with or without good knowledge of COVID-19 after adjustment for possible confounders. A p value less than 0.05 was considered statistically significant. Data management and statistical analysis were performed using SPSS Statistics for Windows, version 25 (IBM Corp., Armonk, N.Y., USA).

## Results

A total of 417 subjects completed the questionnaire. After eliminating 7 incomplete questionnaires, 410 respondents were included in the final analysis (282 females and 128 males). Table 1 shows the social and demographic characteristics of the survey respondents. The mean age of the respondents was 53.8±16.8 years, with 36.6% 65 years old and above and 68.8% female. Among the 410 respondents, 58.5% were categorized as having “good knowledge” for responding to COVID-19 infection for self-reported good or excellent toward all 12 questions in the knowledge part (Part II) of the questionnaire. Table 2 shows the respondents with higher confidence in general questions such as “COVID-19 is an infectious disease transmitted by the respiratory route” and “elderly individuals with COVID-19 infection have a greater risk for severe illness”, and the understanding rates were both above 92% among the total respondents. However, respondents showed lower confidence in relatively detailed questions such as “SARS-CoV-2 can remain viable or infectious on surfaces such as plastics, metal, paper, wood, or glass for 2–5 days”, and the understanding rate was 71.5% among the total respondents.

**Table 1:** Demographic characteristics stratified by age of survey respondents (N = 410).

	All	< 65 years old	≥ 65 years old	p value*
Characteristic	N = 410	n = 260	n = 150	
**Mean age ± SD** (year old)	53.8 (±16.8)	44.0 (±12.5)	71.0 (±5.0)	<0.001
**Sex**				0.69
Male	128 (31.2%)	83 (31.9%)	45 (30%)	
Female	282 (68.8%)	177 (68.1%)	105 (70%)	
**Highest educational level completed**				<0.001
Above high school level	253 (61.7%)	200 (76.9%)	53 (35.3%)	
High school level and below	157 (38.3%)	60 (23.1%)	97 (64.7%)	
**Marital status**				<0.001
Married	263 (64.1%)	141 (54.2%)	122 (81.3%)	
Unmarried and others	147 (35.9%)	119 (45.8%)	28 (18.7%)	
**Occupation**				0.605
Student	21 (5.1%)	11 (4.2%)	10 (6.7%)	
Military, civil service and teacher,	26 (6.3%)	16 (6.2%)	10 (6.7%)	
Medical affair related	81 (19.8%)	49 (18.8%)	32 (21.3%)	
Others	282 (68.8)	184 (70.8%)	98 (65.3%)	
**Medical history**				<0.001
Yes	163 (39.8%)	72 (27.7%)	91 (60.7%)	
No	247 (60.2%)	188 (72.3%)	59 (39.3%)	
**Classification of medical history**				<0.001
Hypertension	110 (26.8%)	46 (17.7%)	64 (57.3%)	
Diabetes	63 (15.4%)	30 (11.5%)	33 (22%)	
**Medical preference for clinic only**				<0.001
Yes	146 (35.6%)	108 (41.5%)	38 (25.3%)	
No	264 (64.4%)	152 (58.5%)	112 (74.7%)	

* The p value was calculated using the Student’s t test for continuous variables or chi-square test for categorial variables to compare between the younger group (< 65 years old) and the elderly group (≥ 65 years old).

The p value < 0.05 represents significance.

**Table 2:** Representative questions summarized for respondents’ knowledge, attitudes, and practices toward coronavirus disease 2019 (COVID-19).

**Knowledge**	**Well understand (%)**	
1. When wearing the medical mask, the colored side should be facing outside and the metal strip should be on the nose.	96.8	
2. One may get sick once becoming exposed to excretions from COVID-19 carriers and then touching his or her own eyes, mouth or nose afterward.	95.9	
3. COVID-19 is an infectious disease transmitted mainly by the respiratory route.	93.9	
4. Elderly individuals with COVID-19 infection have greater risk for severe illness.	92.4	
5. COVID-19 carriers without any symptoms, such as fever or cough, can transmit the disease to others.	89.3	
6. COVID-19 virus can remain viable or infectious on surfaces such as plastics, metal, paper, wood, or glass for 2–5 days.	71.5	
**Attitudes**	**Agree (%)**	**Importance (%)**
1. Maintaining social distance and wearing masks at all times make me feel safer.	98.5	95.9
2. The worldwide COVID-19 condition is severe.	97.6	94.9
3. Avoiding crowded areas or in-person social activities is helpful for disease prevention.	94.9	93.9
4. Following the principles against COVID-19 recommended by the Taiwan CDC is helpful.	93.4	94.1
5. I am willing to receive well-established vaccines, such as influenza or pneumococcal vaccine.	86.6	84.6
6. The COVID-19 condition is severe in Taiwan.	28.3	74.6
**Practices**	**Agree (%)**	
1. I wear a mask at all times when going to crowded areas.	95.6	
2. I wash hand with soap and water or use hand sanitizer more frequently than before.	94.4	
3. I have reduced my visits to crowded areas.	92.4	
4. I will receive the COVID-19 immunization whenever the vaccine becomes available.	76.1	

Regarding attitudes toward COVID-19 disease summarized in Table 2, 97.6% of respondents agreed that the worldwide COVID-19 condition was severe, while only 28.3% of respondents agreed that COVID-19 disease was a severe condition in Taiwan. More than 94% of the respondents agreed that having fewer activities in crowded areas, maintaining social distance and following the principles released from the Taiwan CDC were crucial. Among the total respondents, 86.6% were willing to receive immunization to prevent other kinds of infectious diseases, such as influenza or pneumococcal vaccines.

Regarding practices toward COVID-19, 92.4% of the respondents reported decreasing going to crowded places, 95.6% respondents reported wearing masks at all times when in crowded places and 94.4% respondents increased their frequency of hand washing compared to before the pandemic. Seventy-six percent of the respondents will receive COVID-19 immunization whenever the vaccine is available.

Table 3 shows the association between the respondents’ willingness to immunize for influenza or pneumococcal vaccine in the attitude part and the respondents’ knowledge responding to COVID-19-related questions in the knowledge part of the questionnaire. Demographic characteristics were adjusted for basic confounding factors. There was a significant association between the respondents with good knowledge responding to COVID-19 and the willingness for immunization with influenza or pneumococcal vaccines (OR: 2.85; 95% CI 1.58–5.14) without adjustment. After adjusting for probable confounding factors, sex, age, educational level, medical history and occupation, the respondents with good knowledge responding to COVID-19 had a higher willingness to immunize for influenza or pneumococcal vaccines (OR 3.26; 95% CI 1.74–6.12).

**Table 3:** Odds ratios (ORs) of willingness for influenza or pneumococcal immunization among subjects with good versus poor knowledge in responding to COVID-19.

Poor knowledge N = 170 (41.5%)	Good knowledge N = 240 (58.5%)
Model 1 1.00	2.85 (1.58–5.14) *
Model 2 1.00	3.22 (1.75–5.91)*
Model 3 1.00	3.26 (1.74–6.12) *

*p<0.05.

Model 1: no adjustment.

Model 2: adjusted for sex and age.

Model 3: adjusted for variables in Model 2, plus highest educational level, medical history and occupation.

Those with a score of 1 to 3 points were considered to have poor knowledge and those with a score of 4 to 5 points were good knowledge to respond to COVID-19 in each item. Subjects with all items of good knowledge were regarded as those with good knowledge.

## Discussion

In our survey (data not shown), more than half (58%) of the respondents were presented with good knowledge of their responses to COVID-19. In this good knowledge group, the younger group (<65 years old) comprised 72.5%, and the elderly group (≥ 65 years old) comprised 27.5%. However, the percentages were relatively equal in the “poor understanding of COVID-19 category”, with 50.6% in the younger group and 49.4% in the elderly group. The age discrepancy implied the capacity and ability for information gathering. Younger people were assumed to use mobile devices more frequently than elderly people, so the younger group might have greater accessibility to updated and correct information. The more resources that were available for learning, the more understanding about COVID-19 was assumed.

Participants revealed high confidence in simpler and general questions, such as “COVID-19 is an infectious disease transmitted by the respiratory route” and “elderly individuals with COVID-19 infection have a greater risk for severe illness [22]”, for which the understanding rates in both groups were above 92%. However, respondents showed lower confidence in relatively detailed questions such as “SARS-CoV-2 can remain viable or infectious on surfaces such as plastics, metal, paper, wood, or glass for 2–5 days [3]”, and the understanding rate was 71.5% among the total respondents. This implied that good disease control did not depend on detailed information but simple educational materials and understandable principles released from the government.

Regarding attitudes toward COVID-19 disease, 97.6% of respondents agreed that the worldwide COVID-19 condition was severe, but only 28.3% of respondents agreed that COVID-19 disease was a severe condition in Taiwan. Domestic COVID-19 has been rarely reported in Taiwan, and the confirmed cases were mostly imported. Compared with Indonesian citizens who had a negative attitude toward having to keep a distance of 1.5 meters when in crowds [23], Taiwanese citizens had a positive attitude toward the policies regarding maintaining adequate social distance and wearing medical masks in crowded areas. Furthermore, the Taiwanese government should emphasize disease preventive policies to the public if citizens gradually come to consider COVID-19 infection as a less severe condition due to the rarity of domestic transmission.

**Fig 1:**
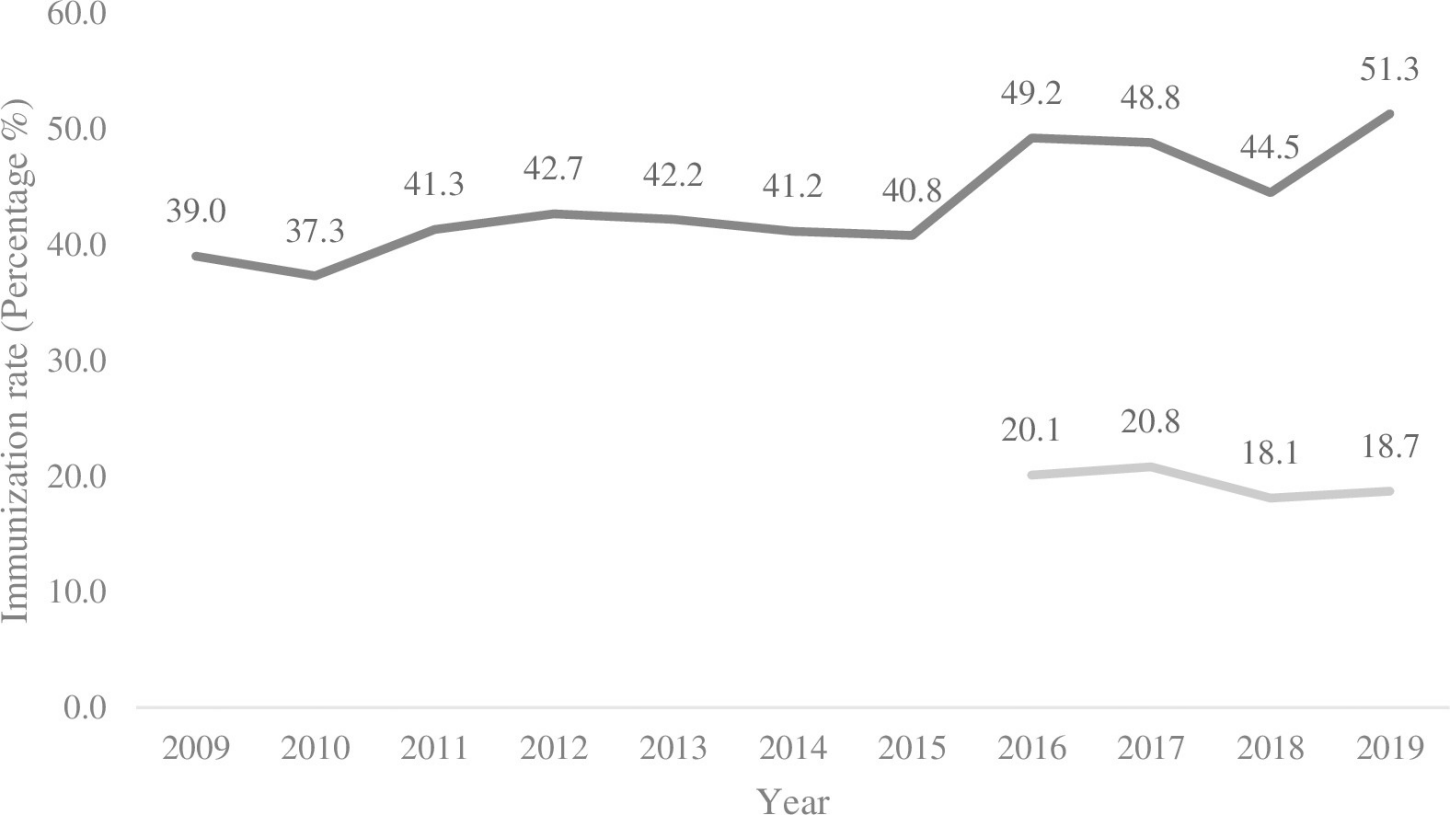
Ten-year influenza vaccine immunization rate in Taiwan. The immunization rate of the influenza vaccine of the Taiwanese population aged 65 and above has been increasing from 39.0% to 51.3% in the past 10 years. The immunization of the influenza vaccine for the 50- to 64-year-old general population has been available since 2016, and the immunization rate has remained at approximately 20%. The black line represents adults aged 65 years old and above and institutional residents. The gray line represents adults aged 50 to 64 years.

In our study, 86.6% respondents agreed that immunizations for other vaccine-preventable diseases, such as influenza and pneumococcal vaccines, would be beneficial. These data reflected the urgency of 1.92 million citizens to receive government-funded seasonal influenza programs from October 5, 2020, a significant increase (26.3%) from 1.52 million doses administered during the same period in last year [24]. Compared with the past 10-year influenza immunization rate of approximately 39% to 51% of the population aged 65 and above and approximately 20% among the healthy population aged 50–64 (Fig 1), citizens’ willingness to be immunized for influenza increased notably in 2020. The willingness to receive influenza and pneumococcal immunizations was significantly associated with respondents’ knowledge responding to COVID-19. After adjusting for age, sex, education level, medical history and occupation, the respondents with good knowledge of COVID-19 appealed to have a significant association with further intention to receive immunization for influenza or pneumococcal vaccine (OR: 3.26; 95% CI 1.74–6.12). In our results, 76% of respondents reported that they will receive COVID-19 immunization whenever the vaccine is available in the future. However, the respondents’ actions were not significantly associated with the respondents’ knowledge of COVID-19. We hypothesized that citizens would be more concerned about the newly developed vaccine than about the well-established seasonal influenza immunization program. Therefore, the government has to emphasize promoting and educating the public regarding the newly designed COVID-19 vaccine.

This study has some limitations. First, the questionnaire was performed in northern Taiwan and the rate of questionnaire incompleteness was 1.7% in this study. However, the unwilling attitude of the populace to fill questionnaire was not evaluated. The small study sample size due to the limited distribution of questionnaire in this cross-sectional study might result in sampling bias. The study duration was relatively short so that these results might not reflect the condition throughout entire COVID-19 epidemics in Taiwan. Second, most of the respondents were female, aged < 65 years, and had higher educational levels; therefore, some of the results of this study might not apply to the entire population. However, we adjusted for biological factors, such as age, sex and medical history, and educational level to minimize this limitation. Last, we did not establish a causal relationship between willingness to be vaccinated and knowledge of COVID-19 due to the cross-sectional design. Nevertheless, few studies have investigated the attitudes, willingness, and awareness of vaccine preventable diseases during COVID-19. We believe that this study can be a reference for government policy consultation in the future.

Participants with good knowledge of COVID-19 are more likely to receive immunization for influenza or pneumococcal vaccines but not COVID-19 vaccines that pass emergency use authorization. Promotion of correct knowledge toward COVID-19 and preparations for immunization are necessary.

## Supporting information

S1 Table Representative questions summarized for respondents’ knowledge, attitudes, and practices toward coronavirus disease 2019 (COVID-19) by age stratification. (DOCX)

S1 Fig Representative questions summarized for respondents’ knowledge, attitudes, and practices toward coronavirus disease 2019 (COVID-19). The parts of knowledge, attitudes, and practices toward COVID-19 in the questionnaire are represented in the separate histograms.
(DOCX)

S1 File Original questionnaire (Traditional Chinese version). (DOCX)

S2 File Original questionnaire (English version). (DOCX)

S3 File Minimal data set. (XLS)
