# Supplementary material for: Impact of Coronavirus Infectious Disease (COVID-19) pandemic on willingness of immunization—A community-based questionnaire study
Source: PLoS One. 2022 Jan 14;17(1):e0262660. doi: 10.1371/journal.pone.0262660 (PMC8759632; doi:10.1371/journal.pone.0262660)
Supplement: S1 Table — (DOCX) [file pone.0262660.s001.docx]

**Table S1**

**Representative questions summarized for respondents’ knowledge, attitudes, and practices toward coronavirus disease 2019 (COVID-19) by age stratification.**

|  | **Total**  **(n=410)** | | **Age**  **< 65 years old**  **(n=260)** | | | **Age**  **≥ 65 years old**  **(n=150)** | | **p value*** |
| --- | --- | --- | --- | --- | --- | --- | --- | --- |
| **Knowledge** | **Well understand (%)** | | | | | | |  |
| 1. COVID-19 is an infectious disease transmitted mainly by the respiratory route. | 93.9 | | 95 | | | 92 | | 0.221 |
| 1. Elderly individuals with COVID-19 infection have greater risk for severe illness. | 92.4 | | 93.5 | | | 90.7 | | 0.303 |
| 1. COVID-19 virus can remain viable or infectious on surfaces such as plastics, metal, paper, wood, or glass for 2-5 days. | 71.5 | | 76.5 | | | 62.7 | | 0.003 |
| 1. COVID-19 carriers without any symptoms, such as fever or cough, can transmit the disease to others. | 89.3 | | 91.9 | | | 84.7 | | 0.022 |
| 1. One may get sick once becoming exposed to excretions from COVID-19 carriers and then touching his or her own eyes, mouth or nose afterward. | 95.9 | | 96.5 | | | 94.7 | | 0.036 |
| 1. It is not suitable for taking the public transportation when one is under quarantine. | 97.1 | | 96.9 | | | 97.3 | | 0.812 |
| 1. One should maintain adequate social distance approximately 1 to 1.5 meters. | 96.8 | | 96.9 | | | 96.7 | | 0.887 |
| 1. Using face mask could reduce the transmission of COVID-19. | 97.3 | | 97.7 | | | 96.7 | | 0.536 |
| 1. When wearing the medical mask, the colored side should be facing outside and the metal strip should be on the nose. | 96.8 | | 97.3 | | | 96 | | 0.467 |
| 1. Handwashing by alcohol sanitizer or water with soap could prevent the transmission of COVID-19. | 96.6 | | 97.7 | | | 94.7 | | 0.104 |
| 1. The 75% v/v alcohol is better than 95% v/v alcohol for disinfection. | 87.8 | | 91.2 | | | 82 | | 0.006 |
| 1. Hypochlorous acid is better used for disinfection for environment than for hand washing. | 87.1 | | 91.2 | | | 80 | | 0.001 |
|  | **Total** | | **Age**  **< 65 years old** | | | **Age**  **≥ 65 years old** | | **p value**  **(agreement)** |
| **Attitudes** | **Agree**  **(%)** | **Importance**  **(%)** | **Agree**  **(%)** | | **Importance**  **(%)** | **Agree**  **(%)** | **Importance**  **(%)** |  |
| 1. Maintaining social distance and wearing masks at all time make me feel safer. | 98.5 | 95.9 | 98.5 | 94.6 | | 99.3 | 98 | 0.308 |
| 1. The worldwide COVID-19 condition is severe. | 97.6 | 94.9 | 98.5 | 95 | | 96 | 94.7 | 0.120 |
| 1. Avoiding crowded areas or in-person social activities is helpful for disease prevention. | 94.9 | 93.9 | 93.8 | 92.7 | | 96.7 | 96 | 0.212 |
| 1. Following the principles against COVID-19 recommended by the Taiwan CDC is helpful. | 93.4 | 94.1 | 91.9 | 93.5 | | 96 | 95.3 | 0.109 |
| 1. I am willing to receive well-established vaccines, such as influenza or pneumococcal vaccine. | 86.6 | 84.6 | 85.4 | 82.3 | | 88.7 | 88.7 | 0.348 |
| 1. The COVID-19 condition is severe in Taiwan. | 28.3 | 74.6 | 27.3 | 71.9 | | 30 | 79.3 | 0.560 |
|  | **Total** | | **Age**  **< 65 years-old** | | | **Age**  **≥ 65 years old** | | **p value** |
| **Practices** | **Agree**  **(%)** | | | | | | |  |
| 1. I wear a mask at all time when going to crowded areas. | 95.6 | | 93.5 | | | 99.3 | | 0.005 |
| 1. I wash hand with soap and water or use hand sanitizer more frequently than before. | 94.4 | | 92.7 | | | 97.3 | | 0.049 |
| 1. I have reduced my visits to crowded areas. | 92.4 | | 92.7 | | | 92 | | 0.798 |
| 1. I will receive the COVID-19 immunization whenever the vaccine becomes available. | 76.1 | | 72.3 | | | 82.7 | | 0.018 |

*The p value was calculated using the chi-square test for the analysis between the younger group (< 65 years old) and the elderly group (≥ 65 years old).

The p value < 0.05 represents significance
