# Supplementary material for: Impact of Coronavirus Infectious Disease (COVID-19) pandemic on willingness of immunization—A community-based questionnaire study
Source: PLoS One. 2022 Jan 14;17(1):e0262660. doi: 10.1371/journal.pone.0262660 (PMC8759632; doi:10.1371/journal.pone.0262660)
Supplement: S1 Fig — The parts of knowledge, attitudes, and practices toward COVID-19 in the questionnaire are represented in the separate histograms. (DOCX) [file pone.0262660.s002.docx]

**S1 Fig**

Representative questions summarized for respondents’ knowledge, attitudes, and practices toward coronavirus disease 2019 (COVID-19)

**S1 Fig 1A** The percentage of participants with well understanding to questions in knowledge part

| Knowledge part | **p value*** |
| --- | --- |
| 1. COVID-19 is an infectious disease transmitted mainly by the respiratory route. | 0.221 |
| 1. Elderly individuals with COVID-19 infection have greater risk for severe illness. | 0.303 |
| 1. COVID-19 virus can remain viable or infectious on surfaces such as plastics, metal, paper, wood, or glass for 2-5 days. | 0.003 |
| 1. COVID-19 carriers without any symptoms, such as fever or cough, can transmit the disease to others. | 0.022 |
| 1. One may get sick once becoming exposed to excretions from COVID-19 carriers and then touching his or her own eyes, mouth or nose afterward. | 0.036 |
| 1. It is not suitable for taking the public transportation when one is under quarantine. | 0.812 |
| 1. One should maintain adequate social distance approximately 1 to 1.5 meters. | 0.887 |
| 1. Using face mask could reduce the transmission of COVID-19. | 0.536 |
| 1. When wearing the medical mask, the colored side should be facing outside and the metal strip should be on the nose. | 0.467 |
| 1. Handwashing by alcohol sanitizer or water with soap could prevent the transmission of COVID-19. | 0.104 |
| 1. The 75% v/v alcohol is better than 95% v/v alcohol for disinfection. | 0.006 |
| 1. Hypochlorous acid is better used for disinfection for environment than for hand washing. | 0.001 |

*The p value was calculated using the chi-square test for the analysis between the younger group (< 65 years old) and the elderly group (≥ 65 years old).

The p value < 0.05 represents significance

**S1 Fig 1B** The percentage of participants answering the agreement to questions in attitude part.

**Figure S1C** The percentage of participants answering the importance to questions in attitude part

| Attitudes |
| --- |
| 1. Maintaining social distance and wearing masks at all times make me feel safer. |
| 1. The worldwide COVID-19 condition is severe. |
| 1. Avoiding crowded areas or in-person social activities is helpful for disease prevention. |
| 1. Following the principles against COVID-19 recommended by the Taiwan CDC is helpful. |
| 1. I am willing to receive well-established vaccines, such as influenza or pneumococcal vaccine. |
| 1. The COVID-19 condition is severe in Taiwan. |

**S1 Fig 1D** The percentage of participants with agreement on questions in practice part

| Practices |
| --- |
| 1. I wear a mask at all times when going to crowded areas. |
| 1. I wash hand with soap and water or use hand sanitizer more frequently than before. |
| 1. I have reduced my visits to crowded areas. |
| 1. I will receive the COVID-19 immunization whenever the vaccine becomes available. |
