# Supplementary material for: Impact of Coronavirus Infectious Disease (COVID-19) pandemic on willingness of immunization—A community-based questionnaire study
Source: PLoS One. 2022 Jan 14;17(1):e0262660. doi: 10.1371/journal.pone.0262660 (PMC8759632; doi:10.1371/journal.pone.0262660)
Supplement: S1 File — (DOCX) [file pone.0262660.s003.docx]

**民眾對於新冠肺炎(COVID-19)態度、認知、行為調查問卷**

| 「您好！隨著臺灣與許多國家交流日益頻繁，前往國外旅遊的人次日漸成長；而這龐大的族群裡，潛藏著許多值得重視的赴外相關健康議題。本問卷想了解您在此次新冠肺炎(COVID-19)流行時，對於相關的健康議題了解與需求程度。問卷採無記名且經保密處理，請放心詳實填寫，謝謝。」 |
| --- |

編號□□□

ㄧ、基本資料

1. 性別：□男 □女 　年齡：________
2. 婚姻狀況：□已婚 □未婚 □其他
3. 教育程度：□不識字 □小學 □國中 □高中 □大學 □研究所或以上
4. 職業：□軍公教 □工商業 □農林漁牧 □自由業 □學生□醫事相關人員□其他
5. 過去疾病病史：□無 □高血壓 □糖尿病 □心臟病 □氣喘 □癌症_____ □其他_____□不清楚
6. 您的運動習慣 □不運動 □偶而運動 □規律運動，每週運動____次，每次_____分鐘
7. 平常就醫習慣：

□診所 □地區/區域醫院 □醫學中心 □無就醫經驗 □其他___________

二、對於新冠肺炎COVID-19的認知情形

| 為了瞭解您在疫情期間對疾病與防疫的認知情形，請您在閱讀各項敘述後，在最適當的□內打勾，答案沒有「對」與「錯」之分。 | | | | | |
| --- | --- | --- | --- | --- | --- |
|  | 非常不了解 | 不了解 | 不確定 | 了解 | 非常了解 |
| 1. 新冠肺炎是一種飛沫傳染為主的呼吸道感染症。------------------ | □ | □ | □ | □ | □ |
| 1. 老年人若罹患新冠肺炎，發生重症風險較高。---------------------- | □ | □ | □ | □ | □ |
| 1. 新冠肺炎病毒在一般氣候下，於物體表面（塑膠、不鏽鋼、金屬、紙、木頭、玻璃）可存活2-5天。-------------------------------------------- | □ | □ | □ | □ | □ |
| 1. 新冠肺炎病毒感染者，沒有症狀時(例如發燒、咳嗽)就能傳染給其他人。----------------------------------------------------------------------- | □ | □ | □ | □ | □ |
| 1. 接觸到新冠肺炎病毒感染者的分泌物再觸摸自己的眼.口或鼻可能會被感染。----------------------------------------------------------------- | □ | □ | □ | □ | □ |
| 1. 進行居家隔離或居家檢疫時，不可搭乘大眾運輸或外出。-------- | □ | □ | □ | □ | □ |
| 1. 無論室內或室外，應維持1-1.5公尺的社交距離。------------------ | □ | □ | □ | □ | □ |
| 1. 使用醫療口罩可以減少新冠肺炎病毒的傳播。----------------------- | □ | □ | □ | □ | □ |
| 1. 配戴醫療口罩時，有顏色面朝外、壓條朝上。-------------------------- | □ | □ | □ | □ | □ |
| 1. 使用酒精乾洗手及濕洗手可以預防新冠肺炎病毒的傳播。------- | □ | □ | □ | □ | □ |
| 1. 消毒用酒精濃度75%的酒精效果優於95%的酒精。---------------- | □ | □ | □ | □ | □ |
| 12. 次氯酸水適合用於環境消毒，不適合用於手部消毒。--------------- | □ | □ | □ | □ | □ |
|  |  |  |  |  |  |

|  |
| --- |

三、對於防疫新生活之相關態度

| 為了瞭解您在疫情期間整體的態度感受，請您在閱讀各項敘述後，在最適當的□內打勾，答案沒有「對」與「錯」之分。 | | | | | | | | | | |
| --- | --- | --- | --- | --- | --- | --- | --- | --- | --- | --- |
|  | 非  常  不  同  意 | 不  同  意 | 中立意見 | 同  意 | 非  常  同  意 | 非  常  不  重  要 | 不  重  要 | 中  立  意  見 | 重  要 | 非  常  重  要 |
|  | 同意程度擇一勾選 | | | | | 重要程度擇一勾選 | | | | |
| 我認為 |  |  |  |  |  |  |  |  |  |  |
| 1. 此次國際的疫情是嚴重的。------------------------------------- | □ | □ | □ | □ | □ | □ | □ | □ | □ | □ |
| 1. 此次臺灣的疫情是嚴重的。------------------------------------- | □ | □ | □ | □ | □ | □ | □ | □ | □ | □ |
| 1. 遵守疫情指揮中心發布的防疫原則是有幫助的。------- | □ | □ | □ | □ | □ | □ | □ | □ | □ | □ |
| 1. 公共場合進行體溫監控是必須的。-------------------------- | □ | □ | □ | □ | □ | □ | □ | □ | □ | □ |
| 1. 「落實手部衛生與咳嗽禮節」是減少呼吸道傳染疾病的好方法。------------------------------------------------------------ | □ | □ | □ | □ | □ | □ | □ | □ | □ | □ |
| 1. 「與他人保持社交距離，全程佩戴口罩」讓我出門較為安心。--------------------------------------------------------------- | □ | □ | □ | □ | □ | □ | □ | □ | □ | □ |
| 1. 保持規律運動，對增強健康免疫力是有幫助的。-------- | □ | □ | □ | □ | □ | □ | □ | □ | □ | □ |
| 1. 出國前，前往旅遊醫學門診對於防疫是有幫助的。----- | □ | □ | □ | □ | □ | □ | □ | □ | □ | □ |
| 1. 疫情期間，減少實體社交活動對防疫是有幫助的。----- | □ | □ | □ | □ | □ | □ | □ | □ | □ | □ |
| 1. 其他傳染病相關疫苗(如:流感疫苗、肺炎鏈球菌疫苗)我會比以前更有意願接種。-------------------------------------- | □ | □ | □ | □ | □ | □ | □ | □ | □ | □ |

四、整體而言，您認為新冠肺炎COVID-19對您的行為是否造成影響？

| 為了瞭解您在疫情期間整體的行為影響，請您在閱讀各項敘述後，在最適當的□內打勾，答案沒有「對」與「錯」之分。 |  |  |  |  |  |
| --- | --- | --- | --- | --- | --- |
|  | 非  常  不  同  意 | 不  同  意 | 中立意見 | 同  意 | 非  常  同  意 |
| 1. 我減少至人潮密集處。----------------------------------------------------------- | □ | □ | □ | □ | □ |
| 1. 我的運動量減少。----------------------------------------------------------------- | □ | □ | □ | □ | □ |
| 1. 我減少急性病的就診。------------------------------------------------------------ | □ | □ | □ | □ | □ |
| 1. 我減少或延後了慢性病回診。-------------------------------------------------- | □ | □ | □ | □ | □ |
| 1. 疫情期間，我改成至診所就診。------------------------------------------------ | □ | □ | □ | □ | □ |
| 1. 疫情期間，我改成至醫學中心就診。------------------------------------------ | □ | □ | □ | □ | □ |
| 1. 未來我會減少出國。-------------------------------------------------------------------- | □ | □ | □ | □ | □ |
| 1. 看到他人戴口罩，會影響我戴口罩的意願。--------------------------------- | □ | □ | □ | □ | □ |
| 1. 我至人潮密集處，都會戴口罩。------------------------------------------------ | □ | □ | □ | □ | □ |
| 1. 我洗手或使用乾洗手次數較疫情前增加。----------------------------------- | □ | □ | □ | □ | □ |
| 1. 我使用線上平台(網路購物、線上開會、視訊聯繫…等)的頻率增加。--- | □ | □ | □ | □ | □ |
| 1. 政府的防疫措施對我的生活造成許多不方便。-------------------------------- | □ | □ | □ | □ | □ |
| 1. 未來若有新冠肺炎疫苗我會接種。------------------------------------------------- | □ | □ | □ | □ | □ |
