# Supplementary material for: Impact of Coronavirus Infectious Disease (COVID-19) pandemic on willingness of immunization—A community-based questionnaire study
Source: PLoS One. 2022 Jan 14;17(1):e0262660. doi: 10.1371/journal.pone.0262660 (PMC8759632; doi:10.1371/journal.pone.0262660)
Supplement: S2 File — (DOCX) [file pone.0262660.s004.docx]

**Questionnaire about knowledge, attitudes and practices toward COVID-19.**

Part I、Demographic data

1. Sex：□male □female
2. Age：_______years old
3. Marital status：□married □unmarried □others
4. Educational level：□illiterate □elementary school □junior high school □senior high school □colleague □master and above
5. Occupation：□military, civil service and teacher □business □farmer □freelancer □student □medical affair related, □others
6. Past medical history：□No □hypertension □diabetes □heart disease □asthma □cancer_____ □others_____□unknown
7. Exercise □No □Occasionally □Regularly, for____time(s) a week and____minute(s) for each time.
8. Meical preference

□local clinic □local hospital □medical center □no medical experience □others___________

Part II、Knowledge of COVID-19

| To understand your knowledge of COVID-19, please check the proper □ after you finished reading the sentences. | | | | | |  |
| --- | --- | --- | --- | --- | --- | --- |
|  | Poorly understand | Not understand | uncertain | understand | Well understand |  |
| 1. COVID-19 is an infectious disease transmitted mainly by the respiratory route. | □ | □ | □ | □ | □ |  |
| 1. Elderly individuals with COVID-19 infection have greater risk for severe illness. | □ | □ | □ | □ | □ |  |
| 1. COVID-19 virus can remain viable or infectious on surfaces such as plastics, metal, paper, wood, or glass for 2-5 days. | □ | □ | □ | □ | □ |  |
| 1. COVID-19 carriers without any symptoms, such as fever or cough, can transmit the disease to others. | □ | □ | □ | □ | □ |  |
| 1. One may get sick once becoming exposed to excretions from COVID-19 carriers and then touching his or her own eyes, mouth or nose afterward. | □ | □ | □ | □ | □ |  |
| 1. It is not suitable for taking the public transportation when one is under quarantine. | □ | □ | □ | □ | □ |  |
| 1. One should maintain adequate social distance approximately 1 to 1.5 meters. | □ | □ | □ | □ | □ |  |
| 1. Using face mask could reduce the transmission of COVID-19. | □ | □ | □ | □ | □ |  |
| 1. When wearing the medical mask, the colored side should be facing outside and the metal strip should be on the nose. | □ | □ | □ | □ | □ |  |
| 1. Handwashing by alcohol sanitizer or water with soap could prevent the transmission of COVID-19. | □ | □ | □ | □ | □ |  |
| 1. The 75% v/v alcohol is better than 95% v/v alcohol for disinfection. | □ | □ | □ | □ | □ |  |
| 12. Hypochlorous acid is better used for disinfection for environment than for hand washing. | □ | □ | □ | □ | □ |  |
|  | | | | | | |

Part III、Attitudes toward COVID-19

| To understand your attitudes and feelings toward COVID-19, please select the proper □ after you finished reading the sentences. | | | | | | | | | | |
| --- | --- | --- | --- | --- | --- | --- | --- | --- | --- | --- |
|  | strongly disagree | disagree | No comment | agree | Strongly agree | very unimportant | unimportant | No comment | important | Very important |
|  | Agreement | | | | | Importance | | | | |
|  |  |  |  |  |  |  |  |  |  |  |
| 1. The worldwide COVID-19 condition is severe. 2. The COVID-19 condition is severe in Taiwan. | □ | □ | □ | □ | □ | □ | □ | □ | □ | □ |
| 1. Following the principles against COVID-19 recommended by the Taiwan CDC is helpful | □ | □ | □ | □ | □ | □ | □ | □ | □ | □ |
| 1. Crowd control measures and body temperature monitoring are necessary in public area. | □ | □ | □ | □ | □ | □ | □ | □ | □ | □ |
| 1. Maintaining hand hygiene and good respiratory etiquette are good ways to reduce respiratory infection. | □ | □ | □ | □ | □ | □ | □ | □ | □ | □ |
| 1. Maintaining social distance and wearing masks at all times make me feel safer. | □ | □ | □ | □ | □ | □ | □ | □ | □ | □ |
| 1. Regular exercise is helpful for keeping good health. | □ | □ | □ | □ | □ | □ | □ | □ | □ | □ |
| 1. Seeking professional consultation in the travel clinic before going abroad is helpful. | □ | □ | □ | □ | □ | □ | □ | □ | □ | □ |
| 1. Avoiding crowded areas or in-person social activities is helpful for disease prevention. 2. I am willing to receive well-established vaccines, such as influenza or pneumococcal vaccine. | □ | □ | □ | □ | □ | □ | □ | □ | □ | □ |

Part IV、Practices toward COVID-19

| To understand your practice or behavioral change toward COVID-19, please select the proper □ after you finished reading the sentences. |  |  |  |  |  |
| --- | --- | --- | --- | --- | --- |
|  | strongly disagree | disagree | No comment | agree | Strongly agree |
| 1. I have reduced my visits to crowded areas. | □ | □ | □ | □ | □ |
| 1. I have reduced the frequency of exercise. | □ | □ | □ | □ | □ |
| 1. I have reduced follow up for acute illness | □ | □ | □ | □ | □ |
| 1. I have reduced or postponed the clinic appointment for chronic illness. | □ | □ | □ | □ | □ |
| 1. I have shifted the medial preference to the local clinic | □ | □ | □ | □ | □ |
| 1. I have shifted the medial preference to the hospital | □ | □ | □ | □ | □ |
| 1. I will avoid going abroad in the future. | □ | □ | □ | □ | □ |
| 1. I will act to wear a face mask when I see other people are waring face masks. | □ | □ | □ | □ | □ |
| 1. I wear a mask at all times when going to crowded areas. | □ | □ | □ | □ | □ |
| 1. I wash hand with soap and water or use hand sanitizer more frequently than before. | □ | □ | □ | □ | □ |
| 1. I used the on-line platform (for shopping, meeting or contacting…etc.) more frequently. | □ | □ | □ | □ | □ |
| 1. I got inconvenience when obeying the principles against COVID-19 recommended by Taiwan CDC. | □ | □ | □ | □ | □ |
| 1. I will receive the COVID-19 immunization whenever the vaccine becomes available. | □ | □ | □ | □ | □ |
